# Rivaroxaban: An Affordable and Effective Alternative in Cancer-Related Thrombosis?

**DOI:** 10.1200/JGO.2015.002527

**Published:** 2016-06-22

**Authors:** Flávia Dias Xavier, Paulo Marcelo Gehm Hoff, Maria Ignez Braghiroli, Ana Carolina Carvalho Rocha Paterlini, Karla Teixeira Souza, Luiza Dib Batista Bugiato Faria, Fernando Sergio Blumm Ferreira, Karime Kalil Machado, Gustavo dos Santos Fernandes

**Affiliations:** **Flávia Dias Xavier**,** Ana Carolina Carvalho Rocha Paterlini**, **Luiza Dib Batista Bugiato Faria**, **Fernando Sergio Blumm Ferreira**, **Karime Kalil Machado**, and **Gustavo dos Santos Fernandes**, Hospital Sírio-Libanês, Brasília; **Paulo Marcelo Gehm Hoff** and **Maria Ignez Braghiroli**, Hospital Sírio-Libanês; and **Karla Teixeira Souza**, Instituto do Cancer do Estado de São Paulo, São Paulo, Brazil.

## Abstract

**Background:**

Venous thromboembolic events (VTEs) are common and potentially fatal complications in cancer patients, and they are responsible for the second most common cause of death. Low molecular weight heparin (LMWH) is the gold-standard treatment, but the costs involved limit its use, especially in developing countries. Recently, the oral anticoagulant rivaroxaban, which directly inhibits factor Xa, was approved for VTE treatment.

**Methods:**

We conducted a retrospective analysis from January 2009 to February 2014 with patients who had cancer and VTE who were receiving rivaroxaban. We compared the efficacy, safety, and cost of rivaroxaban and low molecular weight heparin (LMWH) alone or followed by vitamin K antagonists.

**Results:**

Forty-one patients were identified, with a median age of 62.5 years. The most frequent tumor histology was adenocarcinoma (78%), which was most often found in the colon (26.8%). Most participants had advanced disease and an implanted central venous catheter. Patients’ VTE risk-assessment scores were low (12.5%), intermediate (50%), and high (35.5%). Pulmonary thromboembolism was reported in 41.4% of patients, but inferior limb thrombosis was reported only in 14.6%; 43.9% of patients received enoxaparin before starting rivaroxaban. Rivaroxaban was used for a median time of 5.5 months. Nonmajor bleeding was reported in 12.2% of patients, and rethrombosis was reported in 12.2%. In our study, rivaroxaban was as safe and effective as enoxaparin/vitamin K antagonists (*P* = .54 and *P* = .25, respectively) or LMWH (*P* = .46 and *P* = .29, respectively).

**Conclusion:**

Although our study was a retrospective analysis, our results suggest that in this cohort of oncologic patients, rivaroxaban was safe and effective. Its oral route and lower cost make it an attractive alternative to LMWH, improving management of patients with cancer in low-income countries. Additional studies are necessary to confirm our data.

## INTRODUCTION

Cancer is an independent risk factor for venous thromboembolic events (VTEs).^1-3^ The disease has complex and multifactorial mechanisms that result in the activation of blood coagulation, resulting in a hypercoagulable state that can lead to chronic intravascular coagulation (Table 1).^4^ Tissue factor activity in tumor cells can be enhanced by expression of anionic phospholipids (tableau for assembly of coagulant complexes) or heparanase secreted by tumors (it releases tissue factor pathway inhibitor from endothelial and tumor cells).^5-7^ Tumor cells also modify vascular endothelium by the secretion of proinflammatory (eg, tumor necrosis factor-α and interleukin-1β), proangiogenic (vascular endothelial growth factor), and fibroblast growth factors or by direct adhesion.^4^ Activated neutrophils can carry tumor cells across the endothelial barrier.^4^ Interactions among tissue factor, thrombin, platelets, cancer cells, endothelial cells, macrophages, and smooth muscle cells promote angiogenesis, tumor proliferation, and migration.^4^^,8^^,9^

**Table 1:**
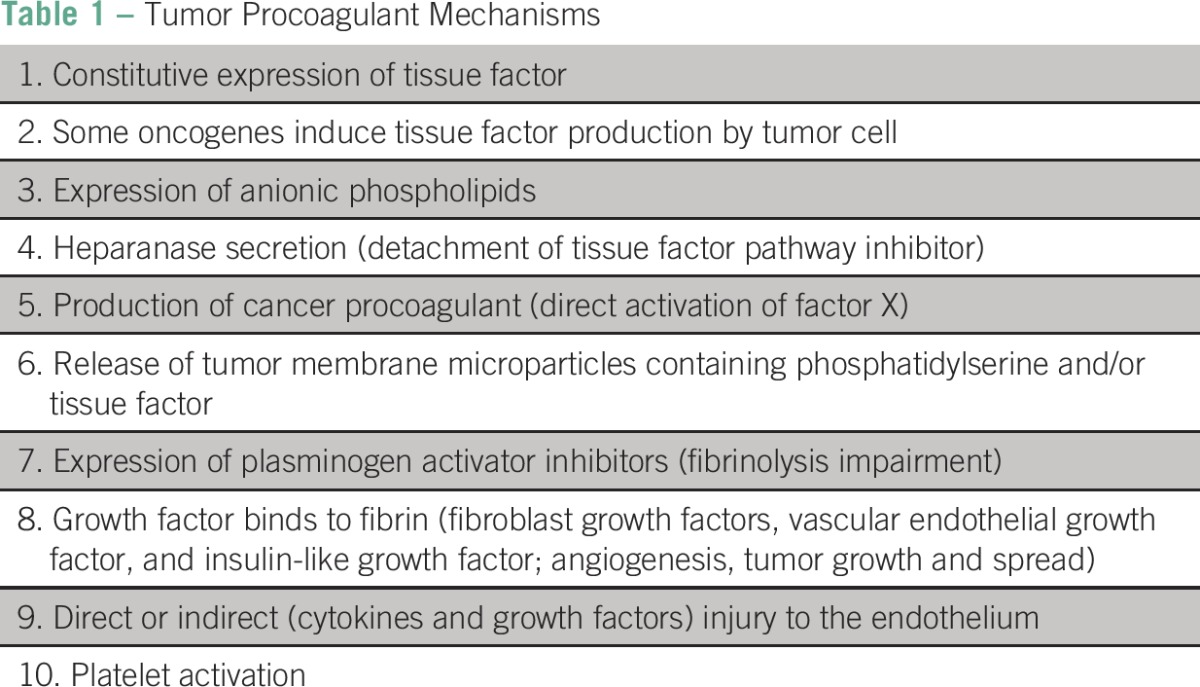
Tumor Procoagulant Mechanisms

Thromboembolic events are common and a potentially fatal complication in cancer patients, corresponding to the second cause of death in these patients and representing an independent mortality risk factor.^10^^,11^ Chemotherapy may result in direct injury to the vascular endothelium, loss of antithrombotic protection mechanisms, increased levels of tissue factor, and secretion of phosphatidylserine and tumor microparticles, favoring hypercoagulability.^12^ However, cancer patients are also more susceptible to bleeding complications, which have been verified in 10% of solid tumors and more frequently in hematologic malignancies.^13^

ASCO supports a predictive model for VTE in outpatients undergoing chemotherapy.^14^^,15^ Established thrombosis in cancer patients is usually treated with low molecular weight heparin (LMWH). ASCO guidelines (2013 to 2014) recommend LMWH for the initial 5 to 10 days of treatment of established deep vein thrombosis (DVT) and pulmonary embolism, as well as for long-term secondary prophylaxis, for at least 6 months.^15,16^

In our practice, many cancer patients do not adapt to treatment with vitamin K antagonist (VKA). Dosing is affected by illness, changes in diet, and numerous interacting medications, and some patients have difficulty adjusting the international normalized ratio (INR) and attending to weekly control of INR; many patients cannot afford long-term treatment with LMWH. Therefore, some patients and some attending physicians opt to use rivaroxaban.^17^ We searched PubMed using the terms cancer, rivaroxaban, and Brazil, and found no published studies on the use of rivaroxaban in cancer patients in Brazil. The objective of our study was to evaluate the efficacy and safety of rivaroxaban in oncologic patients with VTE.

## METHODS

In this retrospective study, we reviewed the electronic medical records of 41 patients with cancer treated at the Oncology Units of Hospital Sírio-Libanês (Bela Vista and Brasilia) from January 2009 to February 2014. Case selection was performed by two nurses who checked the electronic medical records of all scheduled patients who were seen by the attending physicians on a daily basis during the study period. They cross-referenced the patients who had both active cancer and thrombosis and who were also receiving rivaroxaban to make a consecutive list of patients who fit the criteria. We then reviewed the medical records and created a checklist with data about the clinical, oncologic, epidemiologic, and treatment features, as well as thrombotic risk assessment and occurrence of adverse events (bleeding and rethrombosis). The study was conducted according to the Declaration of Helsinki and was approved by a local ethics committee.

Inclusion criteria were age ≥ 18 years; confirmed diagnosis of active cancer; creatinine clearance ≥ 30 ml/min; diagnosis of new-onset VTE confirmed by image examination (Doppler ultrasound, angiotomography, positron emission tomography-computed tomography, or ventilation/perfusion scintigraphy); and an intention to treat with rivaroxaban for at least 3 months. Patients who had previously taken heparin were accepted. All participants consecutively enrolled met eligibility criteria, and none were excluded.

Outcomes were defined as rethrombosis and major (life-threating, red cell transfusion requirement) and nonmajor bleeding during anticoagulation with rivaroxaban. Efficacy was measured by prevention of rethrombosis, and safety was determined by the absence of bleeding.

We performed two-by-two contingency tables (Table 2) on the basis of expected frequencies, assuming that there was no association between event and exposure, to compare our results with previously published data (LMWH alone or followed by VKA)^18^^,19^ with regard to efficacy (prevention of rethrombosis) and safety (absence of bleeding). We calculated relative risk (RR; [a/(a + c)]/[b/(b + d)]), odds ratio (OR; [(a/c)/(b/d)]), and *P* value by Pearson's χ^2^ test (Table 3). A *P* value < .05 was considered statistically significant.

**Table 2:**
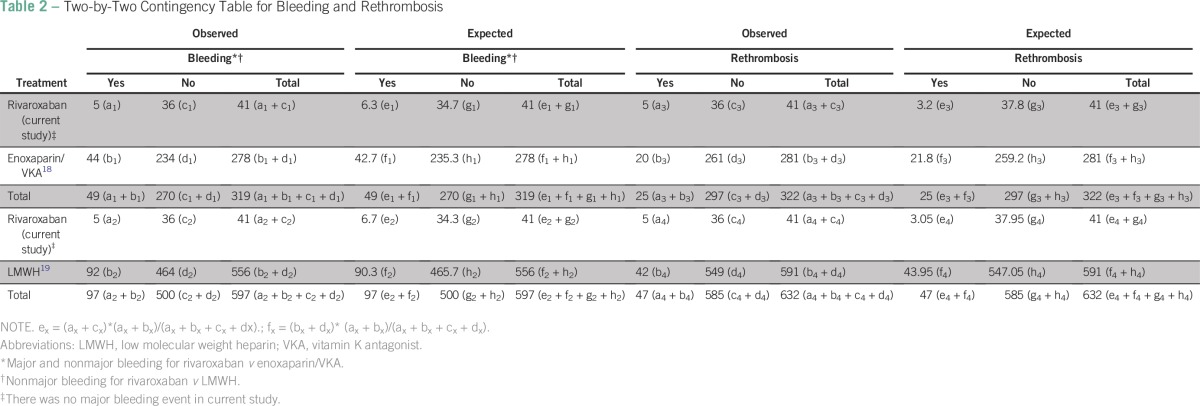
Two-by-Two Contingency Table for Bleeding and Rethrombosis

**Table 3:**
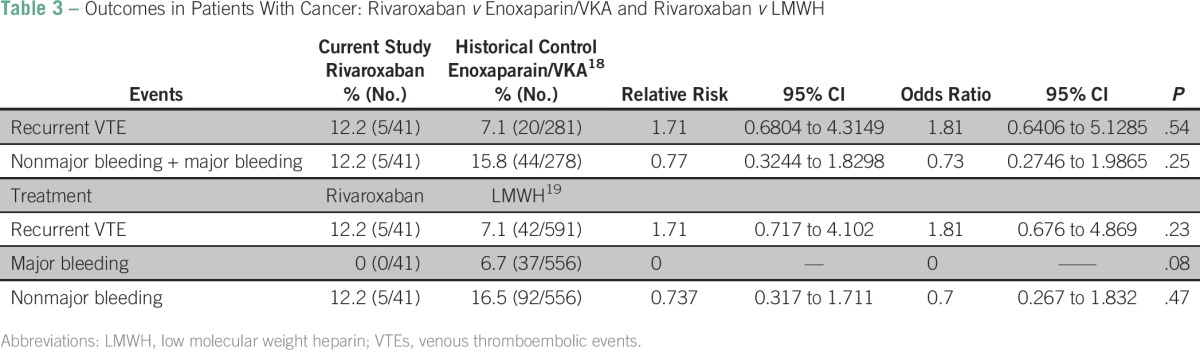
Outcomes in Patients With Cancer: Rivaroxaban *v* Enoxaparin/VKA and Rivaroxaban *v* LMWH

## RESULTS

Patient characteristics are described in Tables 4, 5, and 6. There was a predominance of males (51.2%), and the median age was 62.5 years (range, 18 to 83 years). The most frequent cancer histology was adenocarcinoma (78%), and the most frequent sites of disease were colon (26.8%), pancreas (17.1%), breast (9.7%), and lymph nodes (9.7%). Most patients had advanced disease (87.8%). Other thrombotic risk factors were less common: immobilization (19.5%), recent surgery (7.3%), obesity (2.4%), diabetes (24.4%), hypertension (39%), dyslipidemia (9.8%), smoking (12.2%), active infection (2.4%), and hereditary thrombophilia (2.4%). Only one patient with hereditary thrombophilia had a history of thrombosis. Most patients had an implanted central venous catheter (70.7%). Catheter-associated VTE occurred in 41.4% of these patients. Patients received therapy with tamoxifen (2.4%), bevacizumab (17.1%), or platinum (43.9%).

**Table 4:**
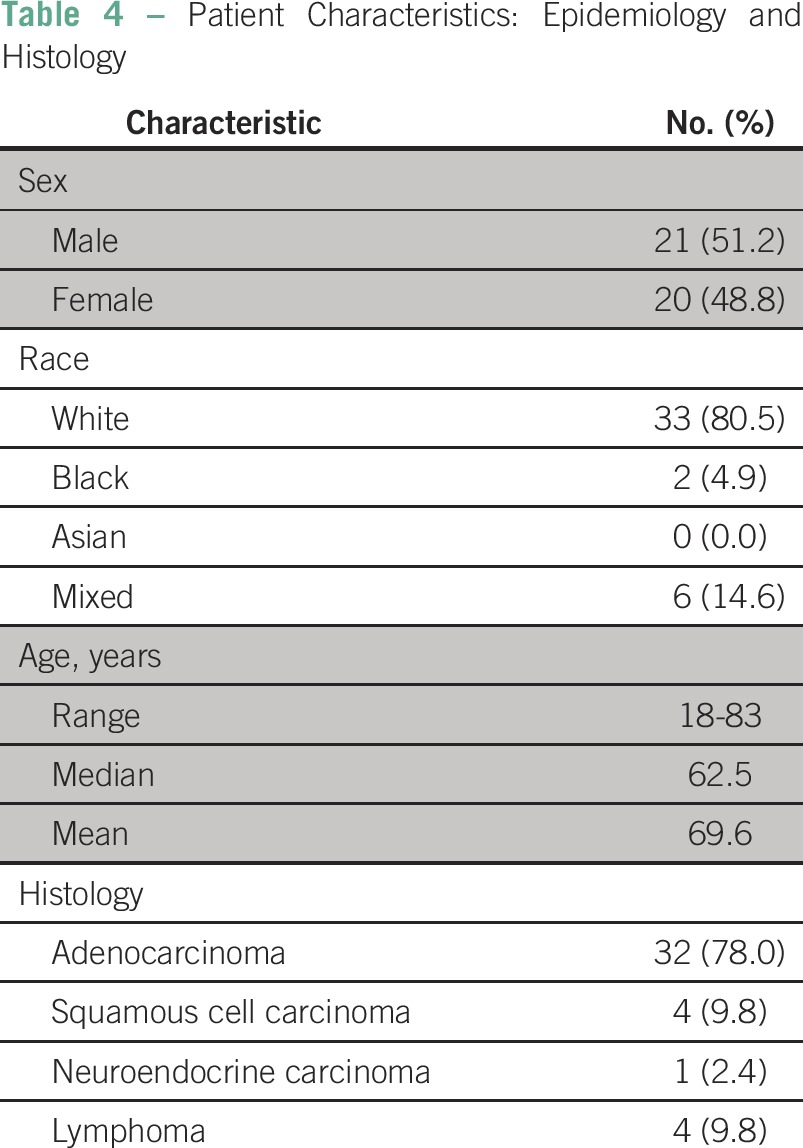
Patient Characteristics: Epidemiology and Histology

**Table 5:**
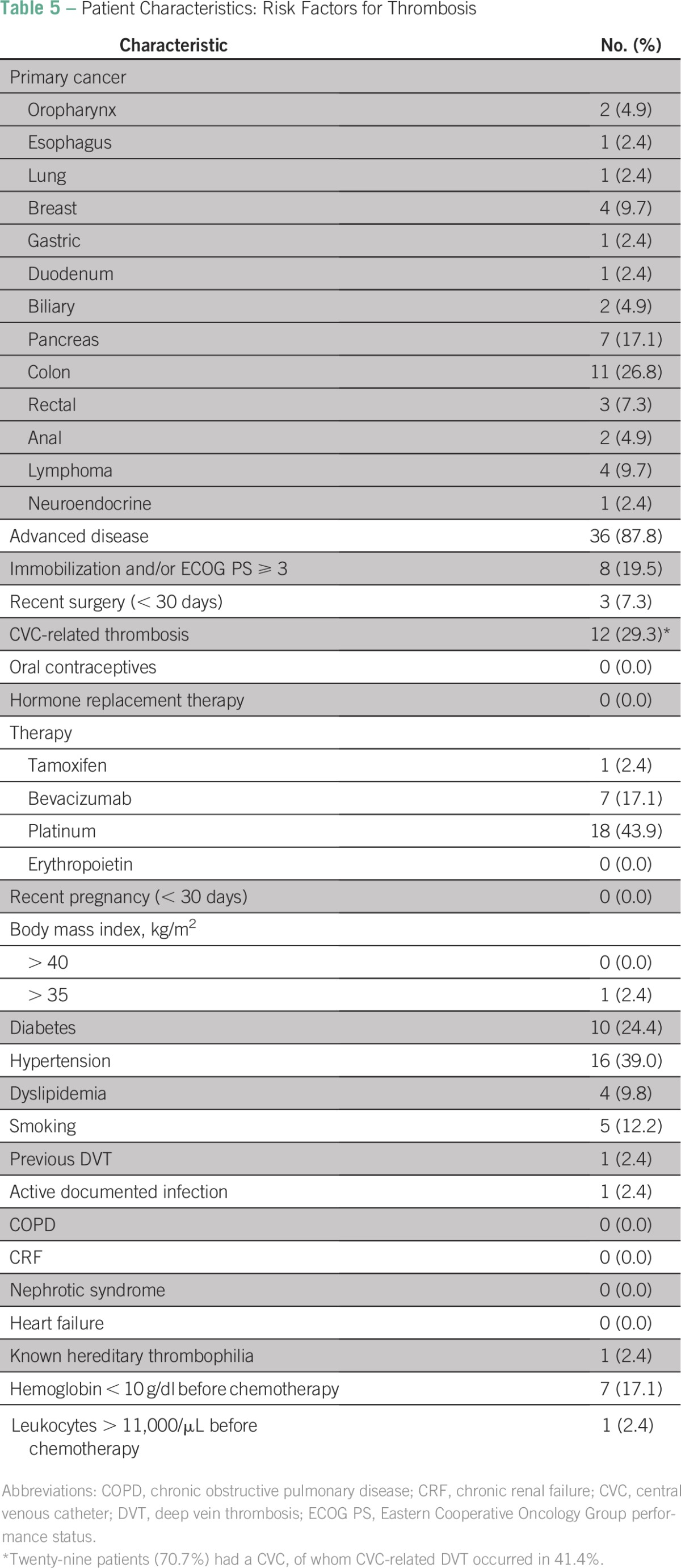
Patient Characteristics: Risk Factors for Thrombosis

**Table 6:**
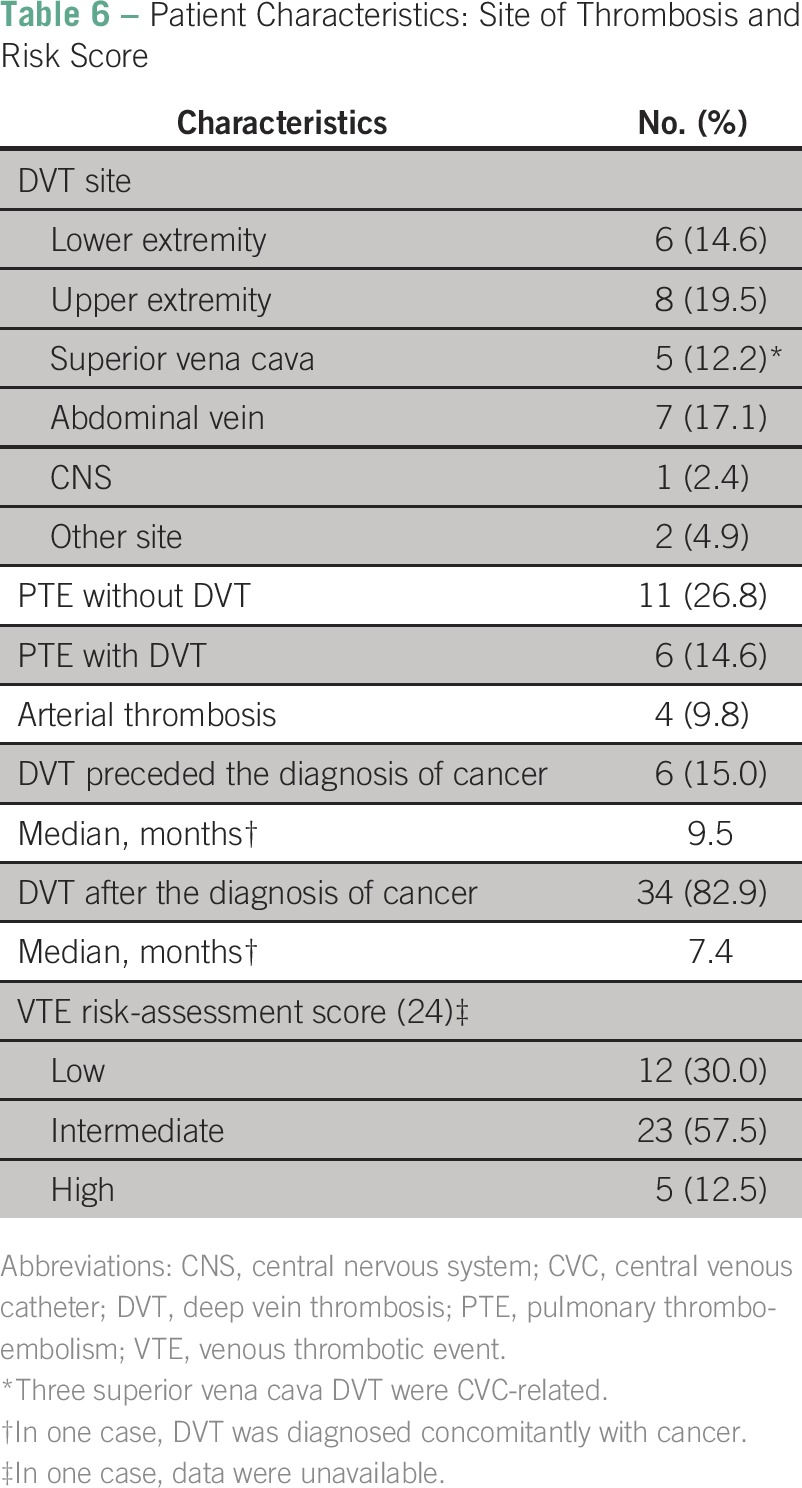
Patient Characteristics: Site of Thrombosis and Risk Score

According to the ASCO VTE risk-assessment score for outpatients, patients were classified as low (30%), intermediate (57.5%), or high (12.5%). Most (> 90%) non–high-risk patients had another VTE predisposing factor in addition to the cancer. Pulmonary thromboembolism was reported in 41.4% of patients, although associated DVT was detected in just 14.6%. Thrombosis of the upper extremity, lower extremity, abdominal vein, and superior cava vena was verified in 19.5%, 14.6%, 17.1%, and 12.2% of patients, respectively. Arterial thrombosis was reported in four patients (9.8%).

Fifteen percent of patients developed thrombosis before the cancer diagnosis, with a median time between thrombosis and diagnosis of 9.5 months. Eighty-three percent of patients developed thrombosis at a median time of 7.4 months after the diagnosis; 43.9% started treatment with LMWH, which was used for a median time of 2.52 months (range, 0.12 to 48.84 months) before starting rivaroxaban. Rivaroxaban was used for a median time of 5.5 months (range, 0.24 to 23.2 months).

The RR (95% CI) and OR for rethrombosis and bleeding with rivaroxaban compared to enoxaparin/VKA were 1.71 (95% CI, 0.6804 to 4.3149) and 1.81; and 0.77 (95% CI, 0.3244 to 1.8298) and 0.73 (Table 3), respectively. In both cases, the 95% CI interval for the RR included 1.0, showing that we could not assume statistical significance. In addition, closer values between RR and OR suggested that the effects of treatment are small and probably not statistically different. Minor bleeding occurred in 12.2% of patients, and just one patient had moderate bleeding. In our study, rivaroxaban was as safe as enoxaparin/VKA (*P* = .25; Table 3). Rethrombosis also occurred in 12.2% of patients during rivaroxaban treatment. In our study, the efficacy of rivaroxaban was not different from traditional enoxaparin/VKA (*P* = .54; Table 3).

We also compared our data with a meta-analysis that included up to five studies on the basis of LMWH alone for treating cancer patients with thrombosis.^19^ In the LMWH group, rates of recurrent VTE, major bleeding, and nonmajor bleeding were 7.1%, 6.7%, and 16.5%, respectively. Comparison of rivaroxaban (the present study) with LMWH did not show a statistical difference in recurrent VTE (*P* = .229), major bleeding (*P* = .08), and nonmajor bleeding (*P* = .466) in cancer patients with thrombosis (Table 3).

## DISCUSSION

Venous thromboembolic disease is a major cause of morbidity and mortality in patients with cancer.^10^ The risk of VTE recurrence is three times greater in cancer patients, despite adequate anticoagulation, than in patients without cancer.^20^ Advanced disease progressively contributes to a patient’s mobility restriction. In our study, advanced disease was common in cancer patients with thrombosis (87.8%), and one fifth had poor performance status (Eastern Cooperative Oncology Group performance status ≥ 3).

Moreover, some therapies, such as tamoxifen (selective estrogen receptor modulator), bevacizumab (antiangiogenic agent, recombinant humanized monoclonal antibody to vascular endothelial growth factor), and platinum were related to increased risk of thrombosis.^21-23^ In our study, 2.4%, 17.1%, and 43.9% of patients were treated with tamoxifen, bevacizumab, and platinum, respectively.

VTE occurred most often in the lungs (pulmonary thromboembolism without DVT, 26.8%), upper extremity (19.5%), and abdominal veins (17.1%). VTE can indicate an occult neoplasm. We found that 15% of patients had developed DVT before the diagnosis of cancer, with a median time of 9.5 months to the onset of the disease.

In our study, colon (26.8%), pancreas (17.1%), and breast (9.7%) cancers and lymphomas (9.7%) were the most prevalent tumors in this population with thrombosis. Pancreatic cancer and hematologic malignancies are traditionally classified as having the highest VTE risk, along with brain, stomach, ovarian, uterus, lung, and kidney cancers.^3,15^ In our study, only 12.5% of patients had a high VTE risk-assessment score for chemotherapy-associated thrombosis, and in four of five patients, primary thromboprophylaxis could have been considered.^14,16^ However, for the majority of patients (87.5%), this score could not predict VTE.

In three meta-analyses, short-term LMWH was more effective than unfractionated heparin,^23-25^ and in the other five, the efficacy was equivalent. LMWH was associated with less bleeding and a significantly reduced mortality rate.^23^ Maintenance with LMWH is recommended as the first choice in both International Society on Thrombosis and Haemostasis^1^ and ASCO guidelines.^14^ In our study, 43.9% of patients started treatment with LMWH, with a median treatment time of 2.52 months, a slightly shorter time than the thrombosis acute phase (first 3 months). After that, the anticoagulant was replaced with rivaroxaban.

Rivaroxaban was the first, oral, direct factor Xa inhibitor. It binds directly and reversibly to factor Xa, inhibiting it competitively. It is 10,000 times more selective for factor Xa than for other serine proteases and requires no cofactors to exert its anticoagulant effect. It inhibits both free and clot-bound factor Xa, as well as prothrombinase activity, thus prolonging clotting times.^26^

Rivaroxaban was recently approved for the treatment of VTE in the general population.^27^ A subanalysis of rivaroxaban use in patients with cancer was recently published.^18^ In that study,^27^ patients with cancer were in the minority (4.7%), and the control group did not include the gold standard of LMWH alone. Therefore, we also compared our data with a meta-analysis^19^ that included up to five studies on the basis of LMWH alone for treating thrombosis and cancer. In our study, we found recurrence rates of 12.2% *v* 7.1% (LMWH/VKA^18^; *P* = .54) and 7.1% (LMWH^19^; *P* = .23); no major bleeding was reported, and nonmajor bleeding rates were 12.2% *v* 15.8% LMWH/VKA^18^; *P* = .25) and 16.5% (LMWH^19^; *P* = .47; Table 3). In our study, rivaroxaban was used for a median time of 5.5 months (0.24 to 23.2 months), and in 56.1% of patients, it was the first line of treatment, most probably related to asymptomatic cases discovered on routine image examinations.

In Brazil, the cost of induction treatment with rivaroxaban 15 mg twice a day for 3 weeks, followed by maintenance with rivaroxaban 20 mg/day for 147 days (6-month treatment) is about R$1,210.92^28^ and is 16 times lower than 6 months of treatment with enoxaparin 60 mg twice a day, which costs approximately R$19,790.40^28^ (in a patient weighing 60 kg). This is just 1.8 times^28^ more expensive than the cost of using 5-day enoxaparin at 60 mg/twice a day, followed by VKA 5 mg/day for 163 days (6-month treatment). However, it has the following advantages: (1) oral route of administration, (2) no need for dose adjustment on the basis of weight or INR monitoring, and (3) no diet restrictions.

In conclusion, it is understanding that rivaroxaban is an alternative treatment for patients with malignancy and VTE. It is cheaper and apparently not inferior to LMWH and should be considered a viable option, especially because the cost of health care has grown substantially. Moreover, its adverse-event profile is consistent with the data published in larger trials. Our study had limitations because it was a retrospective analysis; therefore, randomized trials in patients with malignancy are needed to confirm our findings.
